# The role of glutamate and glutamine metabolism and related transporters in nerve cells

**DOI:** 10.1111/cns.14617

**Published:** 2024-02-15

**Authors:** Dongyang Zhang, Zhongyan Hua, Zhijie Li

**Affiliations:** ^1^ Department of Pediatrics Shengjing Hospital of China Medical University Shenyang Liaoning China; ^2^ Medical Research Center, Liaoning Key Laboratory of Research and Application of Animal Models for Environment and Metabolic Diseases Shengjing Hospital of China Medical University Shenyang Liaoning China

**Keywords:** glutamate metabolism, glutamate transporters, glutamine metabolism, glutamine transporters, nerve cells

## Abstract

**Background:**

Glutamate and glutamine are the most abundant amino acids in the blood and play a crucial role in cell survival in the nervous system. Various transporters found in cell and mitochondrial membranes, such as the solute carriers (SLCs) superfamily, are responsible for maintaining the balance of glutamate and glutamine in the synaptic cleft and within cells. This balance affects the metabolism of glutamate and glutamine as non‐essential amino acids.

**Aims:**

This review aims to provide an overview of the transporters and enzymes associated with glutamate and glutamine in neuronal cells.

**Discussion:**

We delve into the function of glutamate and glutamine in the nervous system by discussing the transporters involved in the glutamate‐glutamine cycle and the key enzymes responsible for their mutual conversion. Additionally, we highlight the role of glutamate and glutamine as carbon and nitrogen donors, as well as their significance as precursors for the synthesis of reduced glutathione (GSH).

**Conclusion:**

Glutamate and glutamine play a crucial role in the brain due to their special effects. It is essential to focus on understanding glutamate and glutamine metabolism to comprehend the physiological behavior of nerve cells and to treat nervous system disorders and cancer.

## Graphical abstract

The glutamate (Glu)‐glutamine (Gln) cycle between neurons and astrocytes, facilitated by cell surface transporters, plays a pivotal role in regulating synaptic glutamate levels, thereby influencing excitatory transmission. Moreover, glutamate serves as a carbon donor to generate α‐ketoglutarate (α‐KG), acts as a nitrogen donor for the synthesis of non‐essential amino acids (NAA), and produces reduced glutathione (GSH), which is essential for the antioxidant system.

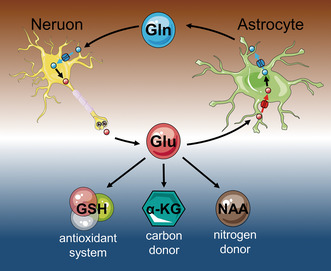

## 1 INTRODUCTION

Glutamine, the most abundant amino acid in the blood, is present in the extracellular fluid of the brain at concentrations of 472 ± 38 μM.^1^ It serves as the primary precursor for glutamate, a crucial excitatory neurotransmitter. Within the brain, glutamate is the most utilized excitatory neurotransmitter in all signal transmission processes.^2^ During synaptic transmission, the release of glutamate from presynaptic neurons via the glutamate–glutamine cycle activates postsynaptic glutamate receptors, thus mediating excitatory transmission.^2^ Simultaneously, this cycle efficiently removes glutamate from the synaptic cleft and maintains a relatively low concentration, preventing excitotoxic damage caused by excessive Ca^2+^ influx into postsynaptic neurons due to the activation of these receptors.^3^ Congenital or acquired defects in synapse formation, glutamate signaling abnormalities, and neural circuit development disorders can lead to severe neurological diseases, including epilepsy, Alzheimer's disease, Parkinson's disease, schizophrenia, and depression.^4^

In addition to its role as a critical excitatory neurotransmitter in the brain, glutamate also serves as an essential carbon and nitrogen donor in cells. The catabolism of glutamine typically occurs in rapidly dividing mitochondria, and dysregulation of intracellular glutamine levels can result in mitochondrial dysfunction, stress response, and ultimately cell death.^5^ Glutamate plays a role in the tricarboxylic acid cycle (TCA) during cellular metabolism, providing the necessary carbon source for the synthesis of biological macromolecules and fatty acids. Glutamate, on the other hand, serves as a vital nitrogen source for the synthesis of nucleotides, glucosamine, and other non‐essential amino acids through transamination or deamination.^6^ Additionally, glutamate is a key component in the synthesis of the antioxidant reduced glutathione (GSH) and forms the GPX4‐GSH antioxidant system with glutathione peroxidase 4 (GPX4), which plays a crucial role in combating oxidative stress and ferroptosis.^7^

This review aims to provide an overview of the glutamate and glutamine transporters and the enzymes responsible for the mutual transformation of glutamate and glutamine within the glutamate–glutamine cycle. Furthermore, it delves into the importance of glutamate and glutamine as nonessential amino acids in neuronal cells, shedding light on their intracellular metabolic behavior.

## 2 GLUTAMATE–GLUTAMINE CYCLE

**FIGURE 1:**
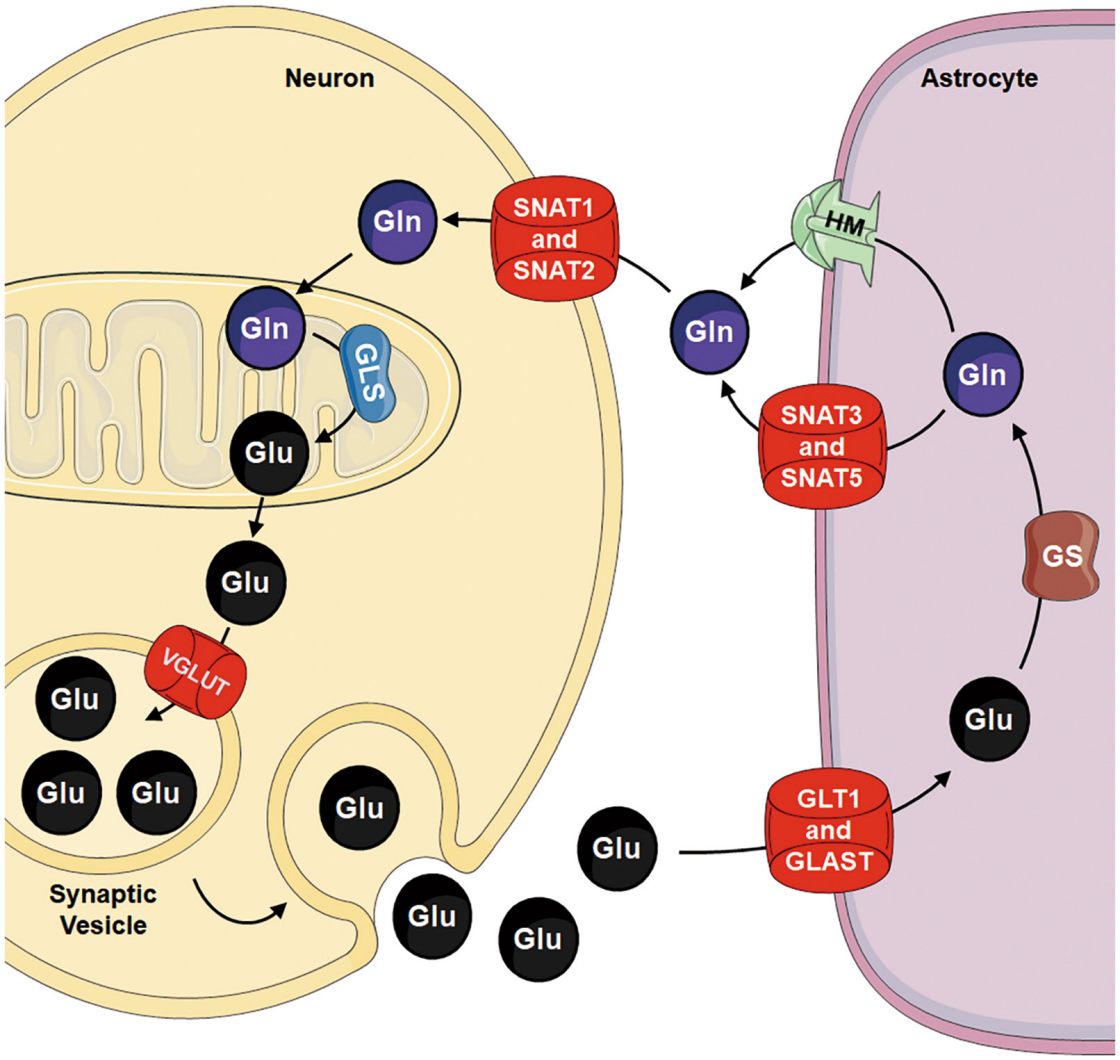
Glutamate–glutamine cycle in nerve cells. Glutamate (Glu) is transported into synaptic vesicles through VGLUTs, and glutamatergic neurons release glutamate to synapses through synaptic vesicles. The postsynaptic glutamate receptors are activated by glutamate, inducing synaptic signal transmission. Most of the glutamate in synapses is transported into astrocytes by GLT and/or GLSAT and converted into glutamine (Gln) in cells by GS. Glutamine in astrocytes is then secreted into synapses through SNAT3, SNAT5, and/or HM. Glutamine is retaken through SNAT1 and/or SNAT2 by neurons, where it is converted into glutamate by GLS.

Glutamate–glutamine cycle suggests that glutamatergic neurons release glutamate to synapses through vesicles, and most of the glutamate in synapses is transported by glutamate transporters on astrocytes and converted into glutamine in cells.^1^, ^8^ Glutamine in astrocytes is then secreted into synapses and retaken by neurons, where it is converted into glutamate (Figure 1).^9^

### 2.1 Neuronal efflux of glutamate

Glutamate in presynaptic terminal neurons is transported into synaptic vesicles by vesicular glutamate transporters (VGLUTs). In mammals, there exist three VGLUT subtypes, namely VGLUT1, VGLUT2, and VGLUT3, encoded by the genes SLC17A7, Slc17A6, and Slc17A8, respectively. These subtypes exhibit largely non‐overlapping distributions within the central nervous system (CNS), forming a complementary glutamate transmission system.^10^ VGLUT1 and VGLUT2 are widely co‐expressed in multiple regions of the CNS, playing predominant roles in central neural circuits.^11^ VGLUT2 has a broader expression pattern and is the sole subtype expressed in the ventral tegmental area and substantia nigra dopaminergic neurons.^12^ VGLUT3 is expressed in a limited population of glutamatergic neurons within the midline thalamic nuclei, cerebral cortex, and inner hair cells of the cochlea. Additionally, it plays a role in non‐glutamatergic neurons, including cholinergic and GABAergic neurons.^11^ VGLUTs demonstrate a relatively low affinity for glutamate, as indicated by a Michaelis constant (Km) ranging from 1 to 2 mM. However, they demonstrate high selectivity for glutamate in comparison to other structurally similar amino acids, such as aspartate or glutamine.^13^ Studies have shown that the concentration of glutamate in synaptic vesicles is about 60 to 120 mM.^13^ VGLUTs are unable to recognize aspartate, whereas Sialin (SLC17A5), which shares a highly similar sequence, can transport both aspartate and glutamate. This discrepancy may be attributed to the presence of arginine in TM7 of the C‐domain at the substrate binding pocket within the VGLUTs, a residue that is highly conserved among the VGLUTs but absent from other SLC17 family members.^14^ Synaptic vesicles use an electrochemical gradient of H^+^ (${∆}_{\mu H^{+}}$) across the vesicle membrane to accumulate and retain neurotransmitters. The H^+^‐ATPase (V‐ATPase) on the vesicle membrane consumes intracellular ATP to transport H^+^ into the vesicle, thereby generating this ${∆}_{\mu H^{+}}$ gradient. The ${∆}_{\mu H^{+}}$ gradient is composed of a chemical H^+^ concentration gradient (ΔpH) and a potential (Δψ), where ${∆}_{\mu H^{+}}=∆\mathrm{pH}+∆\psi$. The process of glutamate entering synaptic vesicles through VGLUTs is mainly driven by Δψ.^15^ Chloride ion (Cl^−^) stimulation, which occurs on either side of the synaptic vesicle membrane, causes VGLUTs to transport glutamate into the synaptic vesicle.^16^ Synaptic vesicles then fuse with the plasma membrane of neurons, releasing glutamate from the vesicles as a neurotransmitter into the synaptic cleft. Following glutamate release, VGLUTs become localized on the plasma membrane. Under certain conditions, such as extensive depolarization or energy depletion, the expression levels and duration of VGLUTs on the plasma membrane may increase.^13^

### 2.2 Glutamate uptake by astrocytes

Astrocytes are equipped with a large number of excitatory amino acid transporters (EAATs), which enable them to rapidly uptake glutamate from synapses. There are five subtypes of EAATs known as EAAT1‐5.^17^ Among these subtypes, EAAT1 (GLAST) and EAAT2 (GLT‐1) are primarily expressed in astrocytes, while EAAT3‐5 are neuron‐specific transporters.^18^ EAATs exhibit a high affinity for Na^+^ and are classified as Na^+^‐dependent EAATs. The transport process involves the uptake of one molecule of glutamate, three Na^+^, and one H^+^ into astrocytes, while simultaneously exporting one K^+^ from astrocytes.^19^, ^20^ Research has indicated that the Km values for glutamate transport into astrocytes, facilitated by EAAT1 and EAAT2 in rats, are 11 and 17 μM, respectively.^21^

GLT‐1 is considered the major glutamate transporter among astrocytic EAATs. It accounts for approximately 1% of the total brain protein in the CNS and is responsible for more than 90% of glutamate uptake.^17^, ^22^ GLT‐1 plays a crucial role in maintaining the homeostasis of the excitatory neurotransmitter glutamate, and as such, it is closely associated with various physiological activities in the brain. Global or astrocytic GLT‐1 deletions result in elevated levels of glutamate, leading to refractory epilepsy and reduced survival, as well as increased susceptibility to acute cortical injury.^3^, ^22^ Deficiency of astrocytic GLT‐1 has been linked to obsessive‐compulsive disorder like repetitive behaviors, such as excessive grooming and twitch‐like behaviors.^23^ Studies using GLT‐1 deficient mice, which express only 20% of normal GLT‐1 levels to simulate chronic GLT‐1 reduction, have shown that these mice exhibited a phenotype resembling attention‐deficit/hyperactivity disorder, including hyperactivity, impulsivity, and impaired memory.^24^ The loss of GLT‐1 can induce anxiety and depression, but the loss of GLT‐1 in different parts leads to different results. Loss of GLT1 in habenular astrocytes has been found to exacerbate depression‐like behavior.^25^ Blocking GLT1 using a GLT1 inhibitor, DHK, in the central amygdala leads to depression and anxiety.^26^ However, down‐regulation of GLT1 by DHK administration in the infralimbic cortex significantly increases glutamatergic neurotransmission, triggering an immediate antidepressant‐like response in rats.^27^ Additionally, GFAP‐positive astrocyte‐specific deletion of GLT1 reduces anxious‐ and depression‐like behavior.^28^

GLAST is highly expressed in astrocytes around the synapses in the cerebellum and cerebral neocortex.^3^, ^29^ Although GLT‐1 is considered to be the main transporter for removing excess glutamate from synapses, GLAST also plays a key role in preventing neuronal damage caused by excitotoxicity. Inhibition of GLAST increases extracellular glutamate levels, leading to excitotoxic neuronal death in mice.^30^ GLAST knockout in mice has been shown to result in increased susceptibility to traumatic brain injury (TBI) and retinal degeneration.^31^ Genetic variants in the GLAST are significantly increased in patients with schizophrenia.^32^ GLAST is expressed in the first 2 weeks after birth, and GLT‐1 is expressed in the first 10–15 days after birth.^33^ DL‐TBOA, a potent excitatory amino acid transporter inhibitor, was injected into mice within 1 week after birth, resulting in cognitive impairment, indicating that GLAST plays an important role in cognitive function.^34^

### 2.3 Synthesis of glutamine in astrocytes

It is estimated that approximately 80% of the glutamate is converted into glutamine through glutamine synthetase (GS; Glutamate ammonia ligase).^3^ GS is an ATP‐dependent enzyme, which catalyzes the reaction of glutamate and ammonia to form glutamine, as follows: $\text{glutamate}+\mathrm{ATP}+{\mathrm{NH}}_{3}\to \text{glutamine}+\mathrm{ADP}+\text{phosphate}$. This process is essential for glutamine synthesis and ammonia clearance.^35^ Research conducted on the spinal cords of 90‐day‐old mice indicates that glutamine synthetase (GS) exhibited a Km value of 2 mM for glutamate.^36^ In the CNS, particularly in the forebrain regions, GS is primarily considered to be an enzyme specific to astrocytes.^37^, ^38^ However, GS expression has also been reported in oligodendrocytes.^39^, ^40^ Studies have demonstrated that the expression of GS in oligodendrocytes is disturbed in chronic neurological diseases in both mouse models and human tissues.^41^

### 2.4 Astrocytic glutamine efflux

In astrocytes, glutamine is mainly released into synapses by astrocyte‐specific amino acid transporters. These amino acid transporters belong to the SLC38 family of the solute carriers (SLCs) superfamily, the sodium‐coupled neutral amino acid transporter (SNAT) family, which is encoded by the gene SLC38A1–11 and has 11 members.^42^ SNAT3 and SNAT5 in the SNAT family, encoded by SLC38A3 and SLC38A5, respectively, are mainly involved in the release of glutamine in astrocytes.^43^, ^44^ SLC38A3 and SLC38A5 belong to system N transporters, which are bidirectional electroneutral glutamine transporters. They transport glutamine into synapses while transporting Na^+^ in the same direction and H^+^ in the opposite direction.^43^, ^45^ In vitro studies of primary astrocyte cultures have shown that system N contributes between 10% and 50% of the total glutamine transport capacity.^46^ Among system N transporters, SLC38A3 is the major transporter for glutamine release in astrocytes, whereas SLC38A5 has a relatively high affinity for serine.^46^ Studies demonstrate that the Km values for glutamine efflux facilitated by SLC38A3 and SLC38A5 are 1.57 and 1.2 mM, respectively.^47^, ^48^ The excretion of glutamine from astrocytes is not only to provide a precursor of neurotransmitters for neurons but also to prevent the accumulation of glutamine from causing damage to astrocytes. At present, there are two theories to explain this damage. According to the Trojan horse theory, glutamine is decomposed into NH_4_^+^ after entering mitochondria, and then reactive oxygen species (ROS) and nitrites are produced, leading to mitochondrial dysfunction and astrocyte swelling.^49^ According to the osmotic gliopathy theory, excessive accumulation of glutamine can inflict osmotic stress on astroglial cells.^50^ Studies have shown that in the mouse model of knocked‐out SLC38A3 activity, associated perivascular astrocytes showed swelling and a decrease in the apparent diffusion coefficient.^51^ A genomic study of 10 patients with developmental and epileptic encephalopathy from seven unrelated families in six different countries found that there are biallelic predicted pathogenic variants in SLC38A3.^52^

Recently, studies on the transfer of glutamine in living cells using glutamine fluorescence probes have shown that glutamine can be released through astrocytic connexin (Cx) hemichannels (HM) to ensure neurotransmitter transmission and cognitive ability.^53^ Cx proteins are a family of proteins that form the structural basis of gap junctions. On the cell membrane Cx monomers are assembled into hexameric connexons, also known as hemichannels (HMs), and two adjacent junction connexons in adjacent cell membranes form gap junction channels (GJCs).^54^ Astrocytes have the highest level of Cx protein expression, and the major Cx proteins are Cx43 and Cx30.^55^, ^56^ GJCs formed by Cx43 and Cx30 between astrocytes form an astrocyte network to transfer or exchange small molecules and ions, such as glucose, amino acids, ATP, and Ca^2+^, which are essential for astrocytic function.^57^ The opening of Cx hemichannels can also release gliotransmitters, including ATP, glutamate, and D‐serine, to maintain neuronal function under physiological conditions.^58^ Studies have shown that astrocyte Cx43 plays an important role in regulating microglial activation, and the loss of Cx43 can significantly improve neuropathic pain caused by spared nerve injury.^59^

### 2.5 Glutamine uptake by neuron

Glutamine secreted by astrocytes into synapses is mainly taken up by amino acid transporters on neurons, including SLC38A1 and SLC38A2. SLC38A1 and SLC38A2 are primarily expressed in neurons in the brain, and their main function is to take up glutamine for glutamatergic and GABAergic neurons.^9^ Both SLC38A1 and SLC38A2 are system A transporters.^60^, ^61^ In neurons, approximately 87% of glutamine is taken up by the system A transporter.^62^ SLC38A1 and SLC38A2 facilitate comparable Km values for glutamine entry into the cell, approximately 0.5 mM.^63^ Meanwhile, studies have shown that glutamine transport through SLC38A2 accounts for about 20% of total glutamine transport.^64^ Similar to SLC38A3 and SLC38A5, SLC38A1 and SLC38A2 also belong to the SNAT family, namely SNAT1 and SNAT2, respectively. In cancer cells, SLC38A1 and SLC38A2 are involved in providing glutamine for glutamine hydrolysis.^65^ Tumor cells can promote the utilization of glutamine through the SNAT family, thereby enhancing the transmembrane transport of glutamine, giving them an advantage in the fierce survival competition with normal cells.^66^ Studies have shown that Down syndrome critical region protein 3 (DSCR3) ensures the uptake and transport of glutamine in glioblastoma multiforme cells by maintaining SLC38A1 protein levels.^67^ Dysfunction of SLC38A2 can also lead to various neurodegenerative diseases, such as Parkinson's disease.^68^ Furthermore, research has revealed that SLC38A1 and SLC38A2 show increased expression levels in tumor cells, promoting tumorigenesis and closely associating with tumor cell proliferation and migration.^69^

### 2.6 Decomposition of glutamine into glutamate

In glutamatergic neurons, glutamine is primarily deaminated in mitochondria by glutaminase (GLS; GA; Glutamine aminohydrolase) to form glutamate, which enters synaptic vesicles to complete the glutamate–glutamine cycle. The reaction process for glutamate formation is $\text{glutamine}+H_{2}O\to \text{glutamate}+NH_{3}$.^35^ Glutamate produced through this reaction serves as a neurotransmitter for glutamatergic neurons and is also the initial step in glutamine catabolism, with GLS acting as a rate‐limiting enzyme in glutamine metabolism.^70^ GLS is mainly encoded by two genes, GLS1 and GLS2. GLS1 encodes renal KGA and GAC isoforms, while GLS2 encodes hepatic GAB and LGA isoforms.^35^ Compared to GLS2, GLS1 is predominantly expressed in the brain.^71^ GLS1 and GLS2 are activated through phosphorylation, earning them the name phosphorylation‐activated glutaminase (PAG).^72^ Within GABAergic neurons, glutamate produced via PAG undergoes conversion to GABA catalyzed by the enzyme glutamic acid decarboxylase. Inorganic phosphate binding is crucial for ensuring the proper orientation of catalytic residues and optimal product release.^73^, ^74^ In the inactive state, GLS1 exists as a dimer, and in the active state, the dimers polymerize with each other to form an active tetramer or a higher polymerization oligomer.^73^ In a study using the GLS inhibitor CB‐839 (Telaglenastat) on three glioblastoma cell lines, it was found that the cell lines exhibited glutamine accumulation, decreased levels of α‐ketoglutarate (α‐KG) and aspartate, and increased levels of acetylation and methylation metabolites. Ultimately, the growth of glioma cells was inhibited.^75^

## 3 INTRACELLULAR METABOLISM OF GLUTAMATE AND GLUTAMINE

### 3.1 Glutamate and glutamine transporter

**FIGURE 2:**
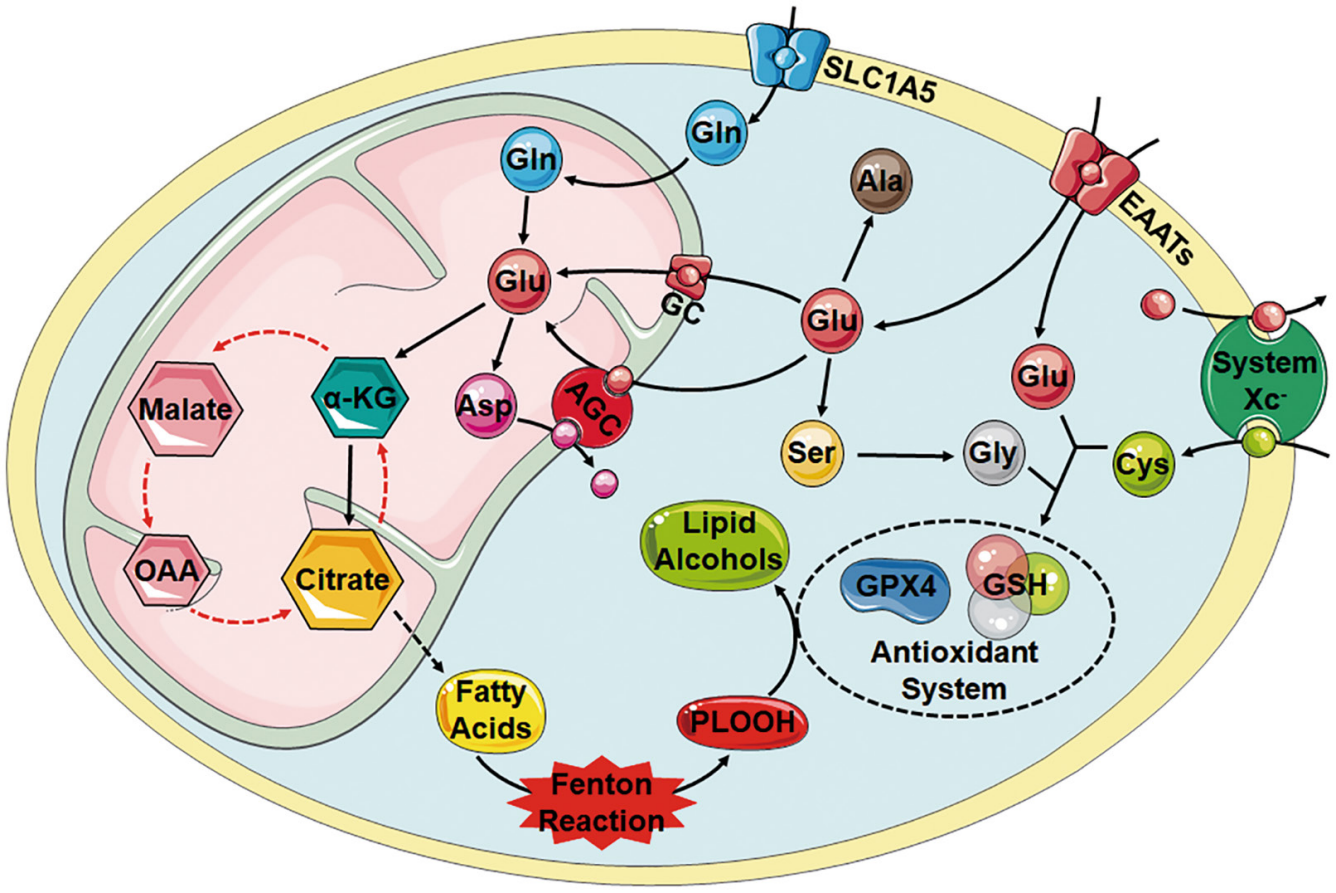
Intracellular metabolism of glutamate and glutamine. In the cytoplasm, glutamate (Glu) generates alanine (Ala) and phosphoserine (pSer) by deamination of glutamate pyruvate transaminase and phosphoserine aminotransferase, respectively. pSer further generates glycine by serine hydroxymethyltransferase. Glutamate, cysteine, and glycine combine to form reduced glutathione (GSH), which helps maintain the content of GPX4 in cells and protects cells from lipid peroxidation. Glutamate is transported into the mitochondria via the GC1 and AGC1 transporters, with the latter closely related to the aspartate–malate shuttle system. The pathway by which glutamine (Gln) enters the mitochondria is not well understood as of now. In mitochondria, glutamate participates in the TCA cycle through the deamination of glutamate dehydrogenase (GLUD) to α‐KG. α‐KG can also generate citrate through the reductive carboxylation (RC) pathway, which is involved in de novo synthesis of fatty acids.

In addition to the role of glutamate as a neurotransmitter in nerve cells mentioned above, it plays an important role as a non‐essential amino acid (Figure 2). There are many kinds of glutamate and glutamine transporters on the surface of the cell and mitochondrial membrane. In addition to the transporters we have mentioned in the glutamate–glutamine cycle section, some other transporters get our attention, such as EAAT3 (SLC1A1), ASCT2 (SLC1A5), SLC25 family, and so on.

#### 3.1.1 EAAT3

EAAT3, also known as excitatory amino acid transporter 1 (EAAC1), is encoded by the SLC1A1 gene and is ubiquitous in the brain and enriched in neurons of the hippocampus and cortex.^76^ EAAT3 is responsible for the uptake of glutamate in 40% of hippocampal neurons and plays a crucial role in maintaining the extracellular glutamate balance in the CNS.^77^ Reports indicate that the Km value for the uptake of [^3^H]glutamate by EAAT3 in glioma cells is 24.3 μM.^78^ Under basal conditions, EAAT3 is primarily intracellular, with only approximately 20% of EAAT3 localized on the cell surface.^79^

EAAT3 expression and function are associated with aging. In the model of LPS‐induced postoperative cognitive dysfunction, the expression of EAAT3 reduced the phosphorylation of GluA1, inhibited the transport of AMPAR to the cell membrane surface, reduced the synaptic density of hippocampal neurons, and then aggravated the LPS‐induced cognitive dysfunction.^76^ In addition to the uptake of glutamate, another notable feature of EAAT3 is its more efficient binding and transport of cysteine, the precursor of neuronal GSH synthesis, compared to astrocytic glutamate transporters (GLAST and GLT1).^80^ Deficiency of EAAT3 results in impaired neuronal GSH metabolism, oxidative stress, and age‐dependent neurodegeneration. Studies have found that the GSH content of hippocampal neurons is reduced, the oxidation level is increased, and the susceptibility to oxidative damage is increased in EAAT3 knockout mice.^80^ EAAT3 can also serve as a potential biomarker of ferroptosis. The main cause of GSH deficiency in neurons was found to be decreased expression of EAAT3. EAAT3 can maintain the activity of the GSH‐GPX4 antioxidant pathway and subsequently scavenge lipid peroxides (LPO). Therefore, EAAT3 plays a crucial role in neuronal defense against ferroptosis.^81^

#### 3.1.2 ASCT2

ASCT2 is a Na^+^‐dependent transmembrane transporter involved in the cellular uptake of neutral amino acids. ASCT2 can transport glutamine in addition to alanine, serine, and cysteine. In cancer cells, ASCT2 is considered to be the main transporter for glutamine uptake.^69^ Examination of human ASCT2, produced and purified from *P. pastoris*, reveals that the wild‐type ASCT2 has a Km value of 35 μM for glutamine transport.^82^ The expression of ASCT2 is significantly up‐regulated in glioblastoma tissues. High expression of ASCT2 can reduce oxidative stress damage in tumors at high metabolic levels by increasing the expression of GPX4, thereby further accelerating the proliferation and progression of tumor cells.^83^ ASCT2 is also associated with inflammatory injury, and its overexpression promotes the activation of the NLRP3 inflammasome, resulting in the cleavage of caspase‐1 and the release of IL‐1b. In vitro and in vivo experiments have demonstrated that reducing astrocytic ASCT2 expression alleviates neuroinflammation in PD models and restores dopaminergic neuronal damage.^84^ The expression of ASCT2 is affected by the MYCN and mTOR signaling pathways. ATF4 and MYCN can synergistically upregulate ASCT2 expression, thereby promoting the invasive progression of MYCN‐amplified neuroblastoma cells.^85^

#### 3.1.3 AGC1 and GC1

There are two carriers for aspartate and glutamate in mitochondria, namely AGC1 (SLC25A12 or Aralar1) and AGC2 (SLC25A13 or citrin).^86^ AGC1 and AGC2 are expressed in excitable and non‐excitable tissues, respectively. AGC1 has a higher expression level in brain and muscle tissues.^87^ AGC is a strict anti‐transport protein. When glutamate and protons are transported from the cytoplasm to the mitochondria, the mitochondrial matrix exports aspartate to the cytoplasm.^86^ AGC plays an important role in the malate–aspartate shuttle system. The malate–aspartate shuttle system compensates for the lack of mitochondrial transporters. Mutations in the SLC25A12 gene lead to AGC1 deficiency. Decreased AGC1 expression can induce oligodendrocyte precursor cell proliferation defects by affecting platelet‐derived growth factor α (PDGFα) and transforming growth factor β (TGFβ), resulting in decreased brain myelination.^87^ AGC1 deficiency also results in decreased levels of N‐acetyl aspartate (NAA) in the CNS, which, in turn, leads to brain myelination decreasing and infantile epileptic encephalopathy.^88^, ^89^ In the presence of N‐acetyl aspartyl‐glutamate (NAAG) synthetase, NAA can be converted to NAAG along with glutamate. NAAG, the most abundant dipeptide in the brain, exhibits significant variations in tissue levels across different regions of the CNS. Notably, spinal cord tissue demonstrates the highest NAAG levels at approximately 2.3 mM, while pituitary tissue shows the lowest levels at around 0.2 mM.^90^ Within neurons, NAAG is primarily concentrated within synaptic vesicles and is released into the synaptic cleft in a Ca^2+^‐dependent manner. Once released, NAAG is hydrolyzed by the highly expressed glial membrane surface enzyme, glutamate carboxypeptidase II, generating NAA and glutamate. Acting as an agonist of the metabotropic glutamate receptor 3, NAAG modulates the release of the excitatory neurotransmitter glutamate. NAAG also serves as a mixed agonist and antagonist of NMDA receptors, influencing the excitability of postsynaptic neurons.^91^ By regulating the release of glutamate, NAAG is believed to play a role in numerous neurological disorders.^90^ In a model of TBI, overexpression of NAAG synthetase effectively increases the concentration of NAAG in local brain areas, exerting a protective effect on neurons.^92^ Alternatively, the NAAG reservoir cycle, involving the conversion from glutamate to NAAG and back to glutamate, provides a mechanism for storing glutamate in cancer cells. When glutaminolysis is inhibited, NAAG can supply glutamate for cancer cells.^93^ Study has found a positive correlation between plasma NAAG concentration and tumor size in vivo, with changes in NAAG concentration preceding changes in tumor size.^94^

In mitochondria, two glutamate carriers, GC1 (SLC25A22) and GC2 (SLC25A18), are also present. In the GC system, glutamate is taken up into the mitochondrial matrix together with protons without the participation of aspartate. It was found that the expression levels of the two kinds of GC in the brain were almost the same.^95^ GC1 has been found to play an important role in cell defense against ferroptosis. It promotes NAPDH synthesis to prevent ferroptosis in cells by mediating GSH production. GC1 promotes the expression of stearoyl‐CoA desaturase (SCD) in cells in an AMPK‐dependent manner, leading to the production of iron‐resistant monounsaturated fatty acids.^96^ Dysfunction of glutamate transporters is directly associated with epilepsy and other neurological deficits.^97^, ^98^ Decreased GC1 activity in astrocytes leads to decreased NAD^+^ and ATP levels, as well as intracellular glutamate accumulation. The accumulation of glutamate in the cytosol of astrocytes leads to the release of glutamate into the synaptic cleft. This glutamate elevation in synaptic strength results in heightened neuronal synchrony, leading to increased amplitude of brain electrical activity oscillations, potentially contributing to the generation of epileptic‐like discharges in patients with GC1 deficiency.^97^, ^99^

### 3.2 Deamination and transamination

Glutamate, which is taken up from the outside of the cell into the mitochondria, and produced from glutamine by the mitochondrial GLS, functions within the cell by deamination and transamination. Glutamate undergoes deamination by GLUD to produce α‐KG, which is an important intermediate in the TCA. TCA is the key cycle of cellular aerobic oxidation, and the deamination of glutamate supplements cellular aerobic oxidation, especially in the case of short‐term lack of glucose supply. This process is also known as glutamate anaplerosis.^7^ Tumor cells have unique metabolic characteristics. They prefer to use glucose for energy production through glycolysis, a phenomenon known as the “Warburg effect.”^100^, ^101^ The metabolic characteristics of the tumor microenvironment (TME) have emerged as a pivotal area of investigation in cancer identification and treatment.^102^ Typically, the predatory uptake of glutamine by tumor cells restricts the availability of glutamine for immune cells, thereby impacting anti‐tumor immune responses.^103^ Mice bearing tumors were administered ^18^F‐Gln to assess the size of the glutamine pool and its uptake in the TME. Subcutaneous tumors demonstrated a pronounced affinity for ^18^F‐Gln.^104^ Furthermore, research has shown that CD40 activation triggers fatty acid oxidation and glutamine metabolism, promoting the epigenetic reprogramming of pro‐inflammatory genes and the emergence of anti‐tumorigenic phenotypes in macrophages.^105^ Compared to glycolysis, the process of the anaplerosis of glutamate plays a crucial role in the survival of tumor cells. In addition to participating in aerobic oxidation in the TCA cycle, α‐KG can also directly produce citrate through a process called RC. Under conditions of long‐term hypoxia or insufficient glucose supply, the pathway of direct citrate production by α‐KG helps maintain the stability of intracellular citrate levels.^106^ Citrate serves as an important precursor for fatty acid synthesis. It is converted to oxaloacetic acid (OAA) and acetyl‐CoA by ATP‐citrate lyase (ACLY), and then used in the de novo synthesis pathway to produce fatty acids.

Another important metabolic pathway of glutamate in cells involves the production of other amino acids, including aspartate, alanine, and serine. This effect is achieved through the catalysis of intracellular transaminases. Glutamate acts as a nitrogen donor in these reactions, transferring amino groups to other substrates to produce α‐KG and another amino acid. Glutamate is converted to aspartate by glutamate oxaloacetate transaminase, to alanine by glutamate pyruvate transaminase, and to phosphoserine (pSer) by phosphoserine aminotransferase.^107^ Serine hydroxymethyltransferase further converts pSer to glycine, which is a component of the intracellular antioxidant GSH. Aspartate serves as the precursor for the synthesis of all nucleotides.^108^

### 3.3 Reduced glutathione

Glutamate exerts an important antioxidant effect in cells, which is realized through the synthesis of GSH. γ‐Glutamyl‐cysteine is synthesized by glutamate‐cysteine ligase. Subsequently, it reacts with glycine under the catalysis of glutathione synthetase to produce GSH.^6^ GSH is the most abundant low‐molecular‐weight thiol in mammalian cells and possesses antioxidant function.^109^ GSH can react with reactive electrophiles and form GSH conjugates through the action of glutathione S‐transferases (GSTs), effectively neutralizing the toxic effects of electrophilic substances. These GSH conjugates are subsequently eliminated from cells by ABC transporters, providing cellular protection. While the overexpression of GSTs enhances the detoxification of anticancer drugs in cancer cells, it also inhibits the activity of ROS that can induce cell apoptosis, thereby increasing drug resistance in cancer cells.^110^ Studies have demonstrated that the heightened activity of GSTs plays a crucial role in counteracting oxidative defense mechanisms.^111^ As a cofactor, GSH also plays an antioxidant role through GPX4, reduces phospholipid hydroperoxide (PLOOH), peroxidized thymine, cholesterol, and fatty acids, and protects cells from oxidative damage.^112^ Hydrogen peroxide has strong permeability and can rapidly react with transition metal (usually iron) to generate hydroxyl radicals, a process known as the Fenton reaction.^113^ The hydroxyl radical possesses a strong oxidative capacity, which can result in the serious accumulation of LPO and ultimately lead to cell death due to iron overload. The level of GSH can maintain the cellular content of GPX4, which is the sole bioactive molecule known to effectively scavenge LPO and PLOOH.^114^ Therefore, glutamate and glutamine play crucial roles in protecting against ferroptosis and oxidative damage.

The synthesis of GSH by glutamate, cysteine, and glycine is primarily regulated by the System Xc^−^ antiporter.^115^ The System Xc^−^ is located on the cell membrane surface and functions as a cystine/glutamate antiporter. It operates independently of Na^+^ but depends on Cl^−^.^116^ The System Xc^−^ antiporter consists of a heavy chain subunit SLC3A2 and a light chain subunit SLC7A11, also known as xCT. These subunits are connected by disulfide bonds. The System Xc^−^ antiporter transports extracellular cystine into the cell and simultaneously exchanges intracellular glutamate outside the cell in a 1:1 ratio.^117^ In the context of System Xc^−^, SLC7A11 serves as the specific main transporter of cystine/glutamate, while SLC3A2 functions as the chaperone protein responsible for maintaining the stability and membrane localization of SLC7A11.^118^ In human retinal pigment epithelial cells, the Km value for glutamate transport facilitated by SLC7A11 is 353 μM.^119^ In normal tissues, SLC7A11 exhibits predominant expression in the brain, while its levels are lower in most other tissues.^120^, ^121^ The transcription of SLC7A11 is regulated by the KEAP1‐NRF2 signaling pathway. Inactivation of KEAP1 stabilizes the NRF2 protein, leading to the up‐regulation of downstream target gene expression controlled by NRF2.^122^ Mutation in KEAP1 and/or hyperactivation of NRF2 result in increased expression of SLC7A11 in cancer cells. This increased expression mediates enhanced intracellular glutamate efflux, leading to increased glutamine dependence and heightened sensitivity to glutaminase inhibition in cancer cells.^123^, ^124^ Studies have demonstrated that activation of the NRF2/SLC7A11/GPX4 signaling pathway can protect neurons from cell damage induced by oxygen–glucose deprivation/reoxygenation (OGD/R), and ultimately prevent the occurrence of ferroptosis. The inhibitor of NRF2, ML385, decreased the protein levels of SLC7A11 and GPX4 in neurons treated with OGD/R.^125^ The function of SLC7A11 is also regulated by other cellular pathways. For instance, p53 down‐regulates SLC7A11, promoting iron‐induced cell death.^126^ Additionally, AMPK mediates the phosphorylation of beclin 1 (BECN1), resulting in the formation of the BECN1‐SLC7A11 complex, and subsequent induction of ferroptosis in vitro.^127^, ^128^

## 4 CONCLUSION

Glutamate and glutamine play a crucial role in the brain due to their special effects. Disordered in the production, accumulation, secretion, and absorption of glutamate and glutamine can lead to corresponding nervous system diseases. Moreover, glutamate and glutamine, as important non‐essential amino acids in cells, are closely related to energy metabolism, lipid metabolism, the production of other amino acids, and the antioxidant effects of cells. Therefore, it is vital to focus on understanding glutamate and glutamine metabolism to comprehend the physiological behavior of nerve cells and to treat nervous system disorders and cancer.

## AUTHOR CONTRIBUTION

Zhijie Li: Supervision, Conceptualization, Writing—Review & Editing, Funding acquisition. Dongyang Zhang: Investigation, Visualization, Writing—Original Draft. Zhongyan Hua: Writing—Review & Editing.

## FUNDING INFORMATION

This work was supported by the “Xingliao Talents Program” of Liaoning Province (XLYC2008010), the National Natural Science Foundation of China (Nos 82272940 and 81972515), the science and technology support program of Liaoning Province (2022JH2/20200075), and the 345 Talent Project and Shengjing Scholar Program of Shengjing Hospital of China Medical University.

## CONFLICT OF INTEREST STATEMENT

The authors have no relevant financial or non‐financial interests to disclose.

## Data Availability

Not applicable.
